# Long-term Prognosis of Patellar Tendinopathy (Jumper’s Knee) in Young, Elite Volleyball Players: Tendon Changes 11 Years After Baseline

**DOI:** 10.1177/03635465241284648

**Published:** 2024-10-22

**Authors:** Håvard Visnes, Lena Kristin Bache-Mathiesen, Tetsuo Yamaguchi, Hans Petter Gilhuus, Knut Robert Hector Algaard, Erling Hisdal, Roald Bahr

**Affiliations:** † Oslo Sports Trauma Research Center, Department of Sports Medicine, Norwegian School of Sport Sciences, Oslo, Norway; ‡ Orthopedic Department, Sørlandet Hospital Kristiansand, Kristiansand, Norway; § Department of Integrated Arts and Social Sciences, Tokushima University, Tokushima, Japan; ‖ Unilabs Radiology, Oslo, Norway; Investigation performed at Oslo Sports Trauma Research Center, Department of Sports Medicine, Norwegian School of Sport Sciences, Oslo, Norway

**Keywords:** knee, jumper’s knee, patellar tendinopathy, prognosis, ultrasound, MRI

## Abstract

**Background:**

The long-term prognosis of jumper's knee and whether structural changes in the patellar tendon persist is unknown.

**Purpose:**

To investigate whether limitations in knee function and structural changes persisted beyond the athletic career of young elite volleyball players.

**Study Design:**

Cohort study; Level of evidence, 2.

**Methods:**

Volleyball players (mean ± SD age, 18 ± 0.8 years) enrolled in 2006-2011 in a prospective cohort study were invited in 2020-2022 to a follow-up study. Participants rated their knee function with the Victorian Institute of Sport Assessment–Patellar Tendon (VISA-P) score (baseline and follow-up) and the International Knee Documentation Committee (IKDC) score (follow-up) and reported if jumper's knee had influenced their decision to retire from sport. Tendon thickness and structural changes were assessed with ultrasound (baseline) and magnetic resonance imaging (MRI) (follow-up) of both patellar tendons.

**Results:**

We included 138 of 143 former athletes (97%) 11.4 ± 1.6 years after their baseline examination. At baseline, 37 persons (52 knees) had developed jumper's knee. At follow-up, participants reported lower knee function scores in knees diagnosed with jumper's knee at baseline than healthy knees (VISA-P scores: jumper's knee, 81 [95% CI, 70-92]; healthy, 90 [95% CI, 86-94]; *P* < .001; IKDC scores: jumper's knee, 82 [95% CI, 75-89]; healthy, 92 [95% CI, 91-95]; *P* < .001). Jumper's knee problems directly caused 7 of the 37 athletes (19%) with jumper's knee at baseline to retire from competitive volleyball. Of the 138 players included, 97 (70%) completed a bilateral MRI examination (194 knees). At follow-up, 38 of the 54 abnormal tendons (70%) had no structural changes (*P* < .001 vs baseline) while 22 of the 140 normal tendons (16%) had developed structural changes. Clinical symptoms were not correlated with tendon structure at follow-up (VISA-P scores for normal tendons: 85 [95% CI, 73-87]; abnormal: 89 [95% CI, 85-92]; *P* = .48).

**Conclusion:**

Jumper’s knee is not a self-limiting condition; volleyball players who had developed jumper's knee during adolescence reported persistent reductions in knee function 11 years later, leading one-fifth to retire from competitive volleyball. Although approximately 70% of tendons with structural changes at baseline were normal at follow-up, there was no clear relationship between structure and function.

The prevalence of jumper's knee is high in sports characterized by high demands on leg extensor speed and power, such as volleyball, basketball, football, and track and field sports.^25^ Elite jumping athletes are the most susceptible,^5,26^ with a point prevalence of jumper's knee as high as 40% to 50% among high-level volleyball players.^26^ Knee pain from jumper's knee can severely limit or even end an athletic career. The mean duration of symptoms among current elite athletes is as high as 32 to 76 months,^26,39^ which means that jumper's knee can be a recalcitrant condition.

Unfortunately, the long-term prognosis for young athletes developing jumper's knee is unknown. Sports medicine clinicians working with young elite jumping athletes are therefore unable to answer the questions often asked by athletes and their parents: Is this a serious injury? If I keep playing, will I have knee problems later in life? Even after I one day quit sport? Currently, the longest follow-up reported in 2 recent systematic reviews on treatment outcomes was 4.3 years.^9,33^ The exception is Kettunen et al^19^ and their case-control study in which they followed 18 athletes with jumper’s knee (3 went on to surgery) and 16 control athletes for 15 years, reporting that symptoms remained after the athletic career was over and that their knee problem caused more than half of those with jumper's knee group to retire from competitive sports.

In many athletes with symptoms of jumper's knee, ultrasound or magnetic resonance imaging (MRI) of the painful tendons will reveal morphological abnormalities, typically localized tendon thickening with hypoechoic areas and increased vascularity.^4,8^ However, as early as 1996, cross-sectional ultrasound studies by Khan et al^20^ and Lian et al^24^ revealed that not only were there symptomatic tendons with normal tendon morphology but also asymptomatic tendons with tendon thickening and hypoechoic areas. These findings have been reproduced in subsequent cross-sectional studies.^6,7,40,42^ However, follow-up studies to identify changes in tendon structure, such as that by Fredberg and Bolvig,^11^ have typically only followed athletes for 1 season. They found that one-third of the abnormal tendons in asymptomatic elite soccer players normalized during the season, while 8% of the normal tendons become abnormal.

In 2006 to 2011, a large cohort of adolescent athletes, students at a 3-year elite sports high school, participated in a prospective study focusing on risk factors for jumper's knee. The findings were published in 4 studies,^16,37,38,40^ documenting that a substantial proportion developed jumper's knee. By following up this cohort 11 years later, we aimed to investigate whether limitations in knee function and structural changes persisted beyond their athletic career, from baseline to follow-up, as well as how many had quit volleyball because of jumper's knee.

## Methods

### Participants and Recruitment Strategy

**Figure 1.**
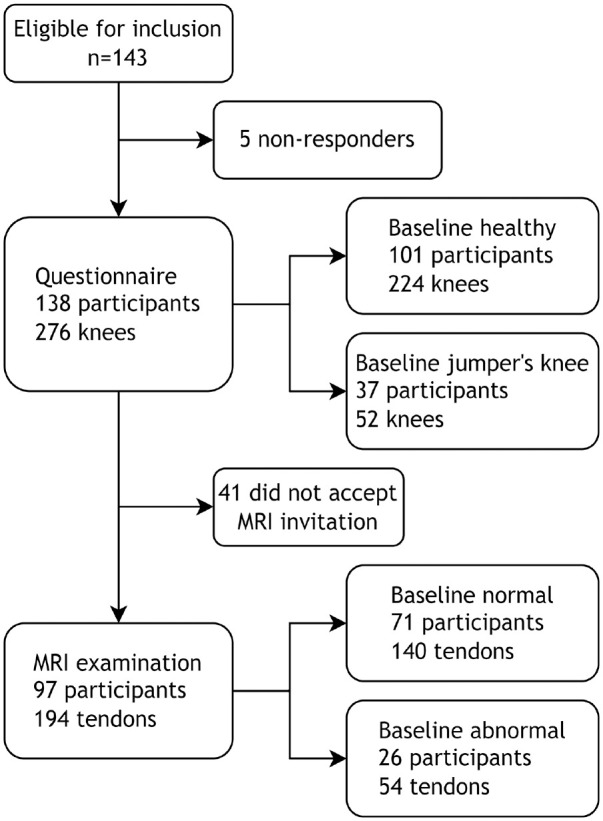
Flowchart of the recruitment process. MRI, magnetic resonance imaging.

Participants (n = 143) from a cohort study during the period of 2006 to 2011 of players at Toppvolley Norge (TVN),^16,37,38,40^ a Norwegian elite sports high school for volleyball, were recruited in 2020 to 2022 to this follow-up study. The study was approved by the regional committee for medical research ethics (No. 107070). The recruitment strategy combined direct mail, a call center, and social media. If respondents consented (N = 138), they were asked to complete a questionnaire and were invited to an MRI examination of both knees, which was completed by 97 participants (Figure 1).

### Baseline Examination

We defined jumper's knee according to the following diagnostic criteria, as in the original cohort^16,37,38,40^: (1) A history of pain in the quadriceps or patellar tendons at their patellar insertions in connection with training or competition and (2) tenderness to palpation corresponding to the painful area. The symptoms had to have been present for ≥12 weeks, and the athlete had to have considered that the symptoms represented a problem.^5,23,25,26,39^ A 5-grade clinical grading system for jumper's knee based on the clinical severity of the problem was published by Roels et al,^35^ but in our analyses we have classified players as having developed jumper's knee or not (yes/no). Their final examination at TVN was used as the baseline classification, but participants who had been diagnosed at any time during high school were still classified as having had jumper's knee.

### Questionnaires

#### The Victorian Institute of Sport Assessment–Patellar Tendon (VISA-P) score

The VISA-P score was designed specifically to quantify knee function in patients with jumper's knee and has been shown to be reliable and valid.^6,7,24^

#### The International Knee Documentation Committee (IKDC)

The IKDC Subjective Knee Form was created as a patient-reported knee-specific assessment of symptoms, function during daily activity, and level of symptom-free sports activity.^13^

#### Treatment Strategy

During high school, treatment was based on the Alfredson eccentric strength training program, as well as jump load adjustments. There was no specific protocol for treatment after patients had left the high school program. The athletes described retrospectively how their jumper's knee problems had been treated, including if they had undergone surgery.

#### Volleyball Participation

Participants were asked to estimate their volleyball activity from baseline to follow-up (hours per week of training, national/junior national team participation, lower level play), and we transformed this information to 3 activity categories: high, high-level play for >7 years; medium, competitive play for 2 to 6 years; low, competitive play for 0 to 2 years.

#### Sports Activity Score

This previously published score (0-100) indicates patients’ general activity level in sports (based on sports type, intensity, and volume) and is not volleyball-specific.^14,17^

#### Reason for Retirement

We also asked if jumper's knee problems directly caused the retirement from volleyball or, if not, if jumper's knee had influenced the patient's decision to retire.

### Ultrasound and MRI Examinations

While attending TVN, participants underwent a bilateral ultrasound scan of their patellar tendon twice a year. Tendon structure at baseline was examined using ultrasound, and the protocol for these athletes has been published in more detail previously.^40^ An experienced orthopaedic surgeon with extensive musculoskeletal ultrasound training and experience, particularly with tendon injuries, who was blinded to the clinical history, conducted all the ultrasound examinations. Two ultrasound machines with similar specifications were used over the study period (GE Vingmed Ultrasound AS: GE Logiq e; 12L-RS probe and GE Logic Book XP; 12L-RS probe). Ultrasound examinations of both the patellar and the quadriceps tendons were carried out with the patient supine and the knee in slight flexion (20°). Tendons with hypoechoic areas were defined as abnormal, and anteroposterior thickness was measured proximally and in the midportion.

At follow-up, the participant underwent standardized conventional MRI scans (1.5 T) of both knees. Two sequences were obtained: T1 turbo spin-echo coronal images and proton density fat-saturated sagittal and axial images, with partition thickness of 3.5 mm and slice thickness with a 3.85-mm interslice gap. Proximal and midportion tendon thickness, tendon abnormalities, and other knee pathology (meniscal, cartilaginous, anterior cruciate ligament [ACL] related, and patellar spur) were assessed. Tendons were classified as abnormal if any of the following structural changes were present: increased signal intensity, interstitial ruptures, partial ruptures of the proximal tendon, or focal thickening.

MRI scans were reviewed fully independently by 2 radiologists (H.P.G., K.R.H.A.) according to predetermined criteria. Because their initial scores for tendon or other knee pathology differed, the case was discussed in a consensus meeting. For tendon thickness, we used the mean between the 2 radiologists’ scores for further analyses. Interrater reliability was assessed with Cohen kappa for categorical variables and intraclass correlation coefficient and Bland-Altman plots for tendon thickness (see Appendix Figure A3 and Table A1, available in the online version of this article, for details).

### Statistical Analysis

Analyses were performed in R 4.2.2 with packages lmtest, mfp, and clubSandwich (R Foundation for Statistical Computing).^18,31,43^

#### Descriptive Statistics

Participant characteristics were calculated as proportions for categorical variables, and mean and standard deviation for continuous variables, after assessing normality. The distribution of baseline and follow-up VISA-P and IKDC scores for jumper's knee and healthy knees was visualized in histograms.

#### Tests and Models

Linear regression was used to determine the association between (1) baseline jumper's knee status and follow-up knee function (VISA-P/IKDC), adjusted for sex; (2) baseline tendon structure and follow-up knee function (VISA-P), adjusted for other knee conditions; (3) tendon thickness and knee function (VISA-P), adjusted for time point (baseline vs follow-up); (4) baseline knee function (VISA-P) and follow-up knee function (VISA-P); and (5) number of abnormal tendons (0, 1, 2) and follow-up knee function (VISA-P).

For (1) and (2), a directed acyclic graph was drawn to identify confounders (see Appendix Figure A1, available online).^36^ Fractional polynomials were used to explore nonlinearity between continuous variables.^1^ For more details, see the Appendix (available online).

To assess change from baseline to follow-up, we calculated the difference in VISA-P and change in, proximal and midportion thickness. The differences were visualized in a histogram for baseline jumper's knee and healthy knees, as well as baseline normal versus abnormal tendons, respectively. A 1-sample *t* test was used to determine whether change was different from zero. In addition, to test whether abnormal tendons at baseline normalized over the follow-up period, a 1-tailed binomial test was run on the percentage of normalized tendons, with 95% Wilson binomial CIs.^2^

We assumed an individual's knees were correlated, and we handled this with cluster-robust standard errors, 95% CIs, and *P* values in all tests and models.^31^ Missing data were negligible and removed from the analyses: 8 baseline VISA-P, 1 follow-up VISA-P, and 2 tendon thickness results.

Data are presented as the mean with standard deviation or 95% CI, unless otherwise noted.

## Results

We included 138 of 143 former athletes (97%) 11.4 ± 1.6 years after their baseline examination (Table 1). Of these, 37 athletes (9 female, 28 male) had developed jumper's knee during their time at TVN. At baseline, lower VISA-P scores were reported by those diagnosed with jumper's knee (n = 52 knees) than with healthy knees (n = 223 knees), with a mean score, respectively, of 78 (95% CI, 73-82) versus 95 (95% CI, 94-96) (*P* < .001). Many continued to play volleyball (36% at high levels of play) during follow-up (Table 1), but there was no difference between groups in current active volleyball participation (*P* = .25) or current sports activity (*P* = .45) Most of the 37 athletes with jumper's knee at baseline reported that they had done specific strength-training programs as treatment (eccentric training on a decline board or heavy slow resistance training), some had additional electrotherapy (shock wave therapy), as well as jump load management. No athlete had undergone surgery for his or her jumper's knee.

**Table 1:** Participant Characteristics at Baseline^*a*^

	Total (N = 138 Persons)	Jumper’s Knee (n = 37 persons)	Healthy (n = 101 persons)
Continuous variables, mean (SD)			
Age, y	18.3 (0.8)	18.5 (0.7)	18.2 (0.8)
VISA-P score (0-100)	91 (15)	78 (21)	95 (9.6)
Height, cm			
Male	188 (6)	188 (7)	188 (6)
Female	172 (7)	172 (8)	172 (6)
Weight, kg			
Male	79 (7)	79 (7.5)	79 (7.4)
Female	68 (8)	66 (9)	68 (7)
Time to follow-up, y	11.4 (1.6)	11.5 (1.6)	11.3 (1.6)
Age at follow-up, y	29.6 (1.8)	30.0 (1.8)	29.5 (1.8)
Sports activity score at follow-up (1-100)	79 (20)	81 (20)	78 (20)
Categorical variables, n (%)			
Sex			
Male	68 (49)	28 (76)	40 (40)
Female	70 (51)	9 (24)	61 (60)
Volleyball activity level after high school			
High	49 (36)	17 (46)	32 (32)
Medium	52 (38)	13 (35)	39 (39)
Low	37 (27)	7 (19)	30 (30)

a VISA-P, Victorian Institute of Sport Assessment–Patellar Tendon score.

### Clinical Outcomes at Follow-up: Did Symptoms Persist?

**Figure 2.**
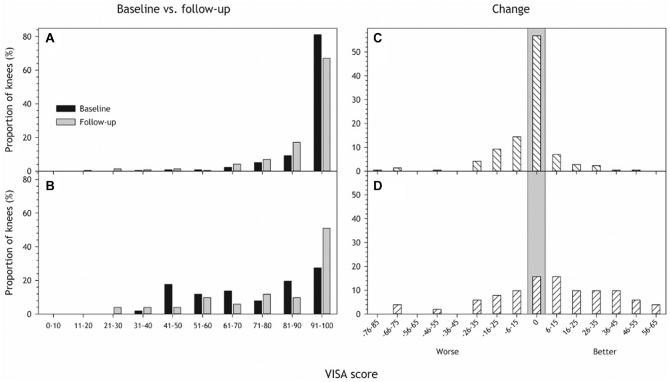
Victorian Institute of Sport Assessment (VISA) score distribution at baseline (black) and follow-up (gray) for (A) healthy and (B) knees diagnosed with jumper's knee at baseline. (C and D) The distribution of change in VISA-P from baseline to follow-up in (C) healthy knees and (D) knees with jumper's knee. A positive number indicates improvement in knee function from baseline to follow-up, a negative number indicates worsening, and zero (vertical gray area) means there was no difference in knee function after 11 years. Participants with missing data (8 missing baseline VISA-P, 1 missing follow-up VISA-P) were removed from the analyses, resulting in 134 participants with 267 knees.

**Figure 3.**
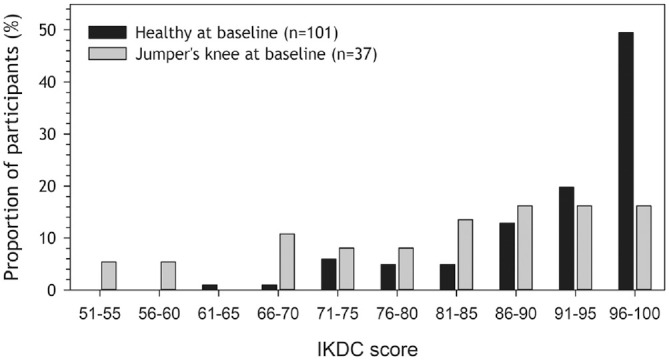
International Knee Documentation Committee (IKDC) score distribution at follow-up for persons with (n = 37; gray) and without (n = 101; black) jumper's knee at baseline.

At follow-up, participants reported lower knee function scores in knees diagnosed with jumper's knee at baseline than healthy knees in both VISA-P scores and IKDC, respectively (81 [95% CI, 70-92]; 90 [95% CI, 86-94]; *P* < .001). This is illustrated in Figure 2, A and B, showing a greater proportion of low VISA-P scores in the jumper's knee group than in the healthy group. The same pattern was observed for IKDC scores (jumper’s knee: 82 [95% CI, 75-89]; healthy: 92 [95% CI, 91-95]; *P* < .001), as shown in Figure 3. The results remained the same after adjusting for sex (see Appendix Table A1, available online).

The change in VISA-P from baseline to follow-up is depicted in Figure 2, C and D. There was some variation with some individuals reporting persistent symptoms, some worsened and some improved. The mean change was 7 points, with a confidence interval overlapping 0 (–3 to 18), meaning we did not detect a significant improvement in VISA-P within the jumper's knee group from baseline to follow-up (*P* = .17). Most of the healthy knees remained the same, but some worsened (Figure 2C), so the overall VISA score also got worse (–3.7 [95% CI, –6.5 to –1.0]; *P* = .008). Low baseline VISA-P was associated with a low follow-up VISA-P (*P* = .011; see Appendix Figure A2, available online).

### Did Jumper's Knee Problems Contribute to Players’ Retiring From Volleyball?

Jumper’s knee problems directly caused 7 of the 37 athletes (19%) with jumper's knee at baseline to retire from competitive volleyball and influenced the decision to quit in 14 additional cases (16% of 86 responses).

### Did Patellar Tendon Structure Normalize From Baseline to Follow-up?

Of the 138 players included, 97 (70%; 194 knees) completed a bilateral MRI examination. A larger proportion of male than female patients underwent MRI examination (53% vs 32%), but there was no difference in the proportion with jumper's knee at baseline (27% vs 25%) or in age distribution. Interrater reliability was weak between the radiologists before the consensus meeting; Cohen kappa ranged between 0.4 and 0.6 for categorical variables (see Appendix Table A1, available online).

Table 2 shows the MRI findings. Of these, 140 tendons were defined as normal on ultrasound at baseline, while 54 had structural changes and were classified as abnormal. At follow-up, 38 of the 54 abnormal tendons at baseline had no structural changes (*P* < .001 vs baseline), while 22 of the 140 normal tendons at baseline (16%) had developed structural changes, but the proportion with structural changes was still greater in the abnormal group (30%; *P* = .047).

**Table 2:** Patellar Tendon Characteristics at Follow-up for Tendons With (Abnormal) and Without (Normal) Structural Changes on Ultrasound Examination at Baseline^*a*^

	Total Knees (n = 194)	Baseline Abnormal (n = 54)	Baseline Normal (n = 140)
Categorical variables, n (%)			
Follow-up abnormal	38 (20)	16 (30)	22 (16)
Increased interstitial signal	21 (11)	9 (17)	12 (9)
Interstitial ruptures	13 (7)	7 (13)	6 (4)
Partial ruptures at tendon insertion	29 (15)	15 (28)	14 (10)
Focal tendon thickness	22 (11)	12 (22)	10 (7)
Meniscal injury	13 (7)	4 (7)	9 (6)
Cartilage injury	8 (4)	3 (6)	5 (4)
ACL injury	00 (0)	00 (0)	00 (0)
Patellar spur	21 (11)	15 (28)	6 (4)
Continuous variables, mean (SD)^*b*^			
Tendon thickness at follow-up, mm			
Proximal	5.3 (1.6)	6.2 (2.1)^*c*^	5.0 (1.2)
Midportion	4.4 (0.9)	4.9 (1.1)^*c*^	4.2 (0.7)
Change in tendon thickness, mm (95% CI)			
Proximal		−0.06 (–0.6 to 0.5)	0.8 (0.6 to 1.1)
Midportion		0.4 (0.06 to 0.7)	0.2 (0.06 to 0.4)

a ACL, anterior cruciate ligament.

b Two tendons had missing thickness data.

c *P* < .05 vs baseline normal tendons.

**Figure 4.**
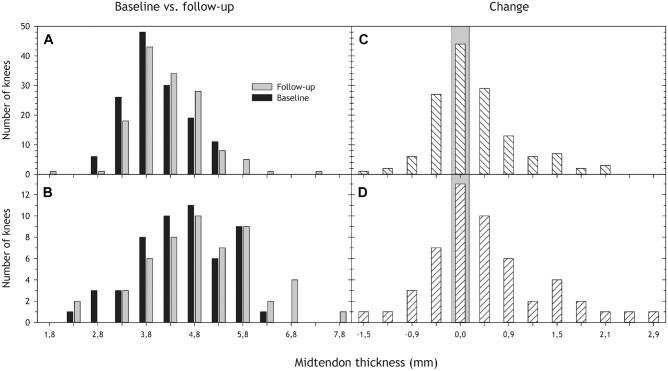
(A and B) Midtendon thickness distribution at baseline (ultrasound; black) and follow-up (magnetic resonance imaging; gray) for tendons with (A) normal and (B) abnormal structure at baseline. (C and D) The distribution of change in midtendon thickness from baseline to follow-up for tendons (C) without and (D) with structural changes at baseline. A positive number indicates increased thickness from baseline to follow-up, a negative number a decrease, and the gray area indicates no change. We removed 1 participant with missing thickness data from the analyses, resulting in 96 participants with 192 knees.

**Figure 5.**
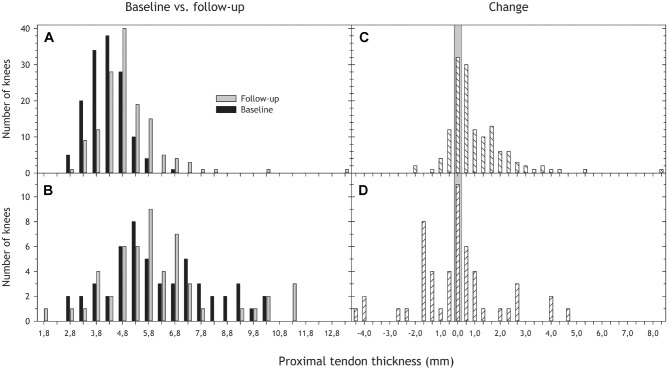
Proximal tendon thickness distribution at baseline (ultrasound; black) and follow-up (magnetic resonance imaging; gray) for tendons with (A) normal and (B) abnormal structure at baseline, as well as change in proximal thickness from baseline to follow-up for tendons (C) without and (D) with structural changes at baseline. See Table 3 for more details.

Midtendon thickness increased slightly from the baseline ultrasound examination to the follow-up MRI location in both groups, tendons with and tendons without structural changes (Table 3, Figure 4). Normal tendons also increased in thickness proximally (Table 3, Figure 5).

**Table 3:** Test of Whether the Mean Change in Tendon Thickness (mm) From Baseline to Follow-up Differs From Zero

Baseline Tendon Structure	Location	Mean Difference	SE	95% CI^*a*^	*P*
Abnormal (n = 52)^*b*^	Proximal	−0.06	0.28	−0.60 to 0.50	.827
	Midtendon	0.38	0.16	0.06 to 0.69	.020
Normal (n = 140)	Proximal	0.81	0.13	0.56 to 1.06	<.001
	Midtendon	0.21	0.07	0.06 to 0.36	<.001

a Cluster-robust CIs.

b Of 54 abnormal tendons, 2 had missing data on tendon thickness.

Interrater tendon thickness variability was on average 0.49 mm (95% CI, –0.96 to 1.94) proximally and 0.12 mm (95% CI, –1.18 to 1.42) in the midtendon, with no clear patterns of bias in the Bland-Altman plot (see Appendix Figure A3, available online). Intraclass correlation coefficient was excellent (0.90) for proximal thickness and good (0.77) for midtendon thickness.

### Were Clinical Symptoms Correlated With Tendon Structure?

Tendon abnormality was not associated with VISA-P score at follow-up (*P* = .48; VISA-P for normal tendons: 85 [73-87]; abnormal: 89 [95% CI, 85-92]). There were too few abnormal tendons with other knee conditions (n = 2) to adjust for these as a potential confounder. A nonlinear relationship emerged between thickness and VISA-P (*P* < .001), showing that both narrow and thick tendons were associated with reduced knee function (see Appendix Figure A4, available online).

## Discussion

The main findings of this prospective cohort show that jumper's knee is not a self-limiting condition; young competitive volleyball players report persistent reductions in knee function from baseline to follow-up 11 years later. Jumper's knee problems directly caused around one-fifth of players with jumper's knee at baseline to retire from competitive volleyball. However, a majority of the structural changes observed at baseline had normalized.

### Clinical Outcomes at Follow-up: Symptoms Persisted

For sports medicine clinicians working with young elite jumping athletes, patellar tendon pain is a common presentation. As stated in the introductory section, these young athletes (and their parents) often ask about the consequences of continuing to play: prognosis and the long-term prognosis. It is well known that some acute knee injuries, especially ACL injuries with associated cartilage and meniscal damage, substantially increase the risk of future knee osteoarthritis. No data exist on the future knee health of athletes developing tendinopathy during adolescence; the longest follow-up reported in 2 recent systematic reviews on treatment outcomes for jumper's knee was 4.3 years.^9,33^

To close this knowledge gap, we conducted the current study, the first prospective, long-term follow-up of a large cohort of young athletes. The main outcome, that symptoms from jumper's knee tend to persist, is perhaps not surprising, considering the high prevalence of jumper's knee among mature elite volleyball players documented in cross-sectional studies. Lian et al,^26^ examining a national elite cohort of 56 players with a mean age of 27 years, found that 45% had current symptoms of jumper's knee. Our findings are also corroborated by data from Kettunen et al,^19^ who reexamined a mixed-sports sample of 18 former athletes, 27 years old at baseline, 15 years after the completion of outpatient treatment, reporting that their symptoms and functional limitations persisted after the athletic career was over. Other sports such as basketball, football, and track and field have similar problems,^5,26,28^ illustrating that jumper's knee represents a problem in sports characterized by high-intensity jumping and sprinting.

In the present study, athletes with jumper's knee reported a deficit in knee function of about 10 points compared with healthy knees on the 100-point VISA-P and IKDC scores at follow-up. This is similar to the report from Kettunen et al^19^ in which adult patients treated for jumper's knee reported more subjective symptoms and functional limitations than control athletes without knee symptoms at baseline, with a mean difference of 11 points on the 100-point Kujala score. Although a difference of around 10 points in these knee function scores may seem trivial, it matches the baseline difference from controls, representing a significant functional limitation, and exceeds the minimal clinically important difference.^3,32^

Notably, in this cohort, jumper's knee also directly caused around one-fifth of the athletes who were symptomatic at baseline to retire from competitive volleyball and influenced the decision to quit in 14 additional cases (16% of 86 responses). Data from Kettunen et al^19^ also showed that 9 of 17 patients were reported to have quit sport because of their knee problem. Still, despite our symptomatic players reporting persistent problems from jumper's knee, they were as physically active with volleyball and other sports as the healthy players. One explanation could be that they just adjusted their activity level to their knee function, because symptoms tend to flare up when volume and intensity increase.^10^

In other words, chronic jumper's knee is not a completely debilitating condition. For most participants, the knee allowed for an active lifestyle, albeit with some reduced function. Still, we would argue that adolescents presenting with recent onset of jumper's knee should be informed that this is not a self-limiting condition; and a treatment plan, usually requiring time off from competitive sport, aiming for complete resolution of symptoms should be initiated without delay.

### Weak Relationship Between Patellar Tendon Structure and Symptoms

Our data showed that 70% of abnormal tendons had normalized during the 11-year observation period. Despite this, symptoms persisted. Also, 16% of normal tendons had developed structural changes. There exists no comparable study investigating changes in the patellar tendon on ultrasound or MRI over a 10-year period. However, cross-sectional studies show that abnormal tendons are common in sports. Walczak et al^41^ found that 89% of professional basketball players have ≥1 MRI abnormalities within their knees. Levin et al^22^ examined 100 consecutives knees of patients referred to an MRI institute for knee pain and concluded that there was a high proportion of increased signal within the proximal patellar tendon without any associated clinical signs or symptoms or signs of tendinopathy. Hamilton and Purdam^15^ described the hypoechoic lesion seen in the patellar tendon in athletes with jumper's knee as the result of adaptation or partial adaptation of the proximal patellar tendon to a compressive load. Golman et al^12^ compared 85 patients with patellar tendinopathy with 86 matched controls. The spectrum of pathology included similar findings to our study.

Previous prospective studies on structural tendon changes are limited to 1 season (<1 year). Fredberg and Bolvig^11^ found that one-third of the abnormal tendons in 49 asymptomatic elite soccer players normalized during the season, while 8% of the normal tendons became abnormal. Pappas et al^29^ examined 24 asymptomatic college basketball players with MRI before and after a season. Preseason scans revealed ≥1 structural abnormalities in all 24 asymptomatic knees. Patellar tendinopathy was the most common finding (83%), and there were no changes when reexamined (21 players) after the season.

Clinical symptoms were not correlated with tendon structure at follow-up in our cohort. This finding is in line with ultrasound studies from the early 1990s by Khan et al^20^ and Lian et al,^24^ as well as a recent study by Rabello et al.^34^ Screening of asymptomatic athletes has also clearly shown that changes found on ultrasound or MRI do not correlate with clinical symptoms.^30^ In addition, postoperative tendon imaging (ultrasound or MRI) does not correlate with clinical outcome and is not able to differentiate good from bad results.^21^ Consequently, imaging results of patients with patellar tendinopathy are not in themselves decisive for prognosis.^30^

### Strengths and Limitations

The main strength of this study was that we followed a cohort of 138 volleyball players for 11 years with a response rate of 97%. A representative sample of 70% also completed a bilateral MRI examination. The main limitation is that we could not perform a clinical examination at follow-up, as was done at baseline. This means that we cannot rule out that other conditions than jumper's knee may have caused the limitations in knee function reported. Still, there was no group difference in the proportion with cartilage or meniscal injury, and none had undergone ACL reconstruction or surgery for jumper's knee. It therefore seems reasonable to assume that the tendon pain was the main cause of the group differences and persistent symptoms observed.

Another limitation is that the data on volleyball activity and treatment strategy during the 11-year follow-up period were collected retrospectively, with a substantial risk of recall bias. We therefore categorized volleyball activity into 3 groups, which lowered precision but also provided better accuracy. Different patterns of volleyball activity and participation may be a confounding factor for knee function that our data were not sufficiently detailed to provide. We were not able to record a precise description of the treatment received after high school (protocols, duration, etc), but the fact that the athletes received treatment from different providers where they lived and played during this 11-year period increases the external validity of the main finding, that symptoms and tendon changes tended to persist.

This study compared ultrasound data with MRI data collected 11 years later. Interrater reliability of the 2 blinded radiologists was good for the tendon thickness measures, and we used a consensus approach for the categorical measures where the initial reliability was lower. Also, because the modality was different, whether a direct comparison of the data is valid may be questioned. Nishida et al^27^ evaluated 65 elite university athletes (130 knees) using both ultrasound and MRI and found that MRI measured tendons 0.4 to 0.5 mm thicker than ultrasound. This suggests that our finding of increased tendon thickness from baseline to follow-up in 3 of 4 measurements might represent a systematic difference between the 2 modalities. Warden et al^42^ examined 30 participants with clinically diagnosed patellar tendinopathy and 33 activity-matched, asymptomatic participants with ultrasound and MRI. Khan et al^20^ found no statistically differences between the mean anteroposterior dimension of the tendon measured with ultrasound and those measured with MRI. They concluded that both MRI and ultrasound were accurate in confirming a pathological tendon, and our data should therefore also be comparable. Also, the design of the study is a comparison with the “control group,” the athletes without tendon changes at baseline, so if there is a systematic difference between MRI and ultrasound, this is accounted for in this comparison.

## Conclusion

Jumper’s knee is not a self-limiting condition; volleyball players who had developed jumper's knee during adolescence reported persistent reductions in knee function 11 years later, leading a substantial proportion to retire from competitive volleyball. Although approximately 70% of tendons with structural changes at baseline were normal at follow-up, there was no clear relationship between structure and function. Athletes presenting with early symptoms of jumper's knee should receive appropriate treatment without delay to prevent prolonged, recalcitrant symptoms and reduced knee function.

## Supplemental Material

sj-pdf-1-ajs-10.1177_03635465241284648 – Supplemental material for Long-term Prognosis of Patellar Tendinopathy (Jumper’s Knee) in Young, Elite Volleyball Players: Tendon Changes 11 Years After Baseline Supplemental material, sj-pdf-1-ajs-10.1177_03635465241284648 for Long-term Prognosis of Patellar Tendinopathy (Jumper’s Knee) in Young, Elite Volleyball Players: Tendon Changes 11 Years After Baseline by Håvard Visnes, Lena Kristin Bache-Mathiesen, Tetsuo Yamaguchi, Hans Petter Gilhuus, Knut Robert Hector Algaard, Erling Hisdal and Roald Bahr in The American Journal of Sports Medicine
