# Supplementary material for: Long-term Prognosis of Patellar Tendinopathy (Jumper’s Knee) in Young, Elite Volleyball Players: Tendon Changes 11 Years After Baseline
Source: Am J Sports Med. 2024 Oct 22;52(13):3314–23. doi: 10.1177/03635465241284648 (PMC11542324; doi:10.1177/03635465241284648)
Supplement: sj-pdf-1-ajs-10.1177_03635465241284648 – Supplemental material for Long-term Prognosis of Patellar Tendinopathy (Jumper’s Knee) in Young, Elite Volleyball Players: Tendon Changes 11 Years After Baseline [file sj-pdf-1-ajs-10.1177_03635465241284648.pdf]

Appendix

Figures

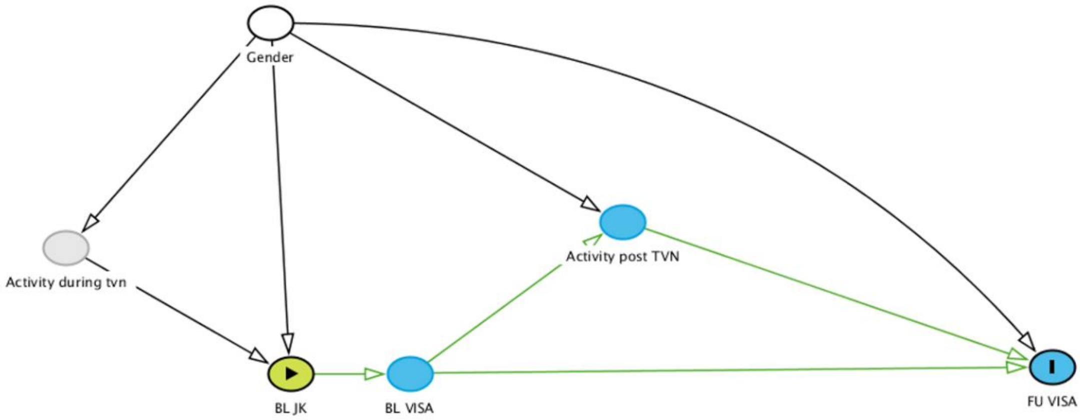

Figure A1. Hypothetical relationship between jumper's knee at baseline (BL JK) and follow up VISA-score. If adjusted for gender, the main source of confounding should be accounted for.

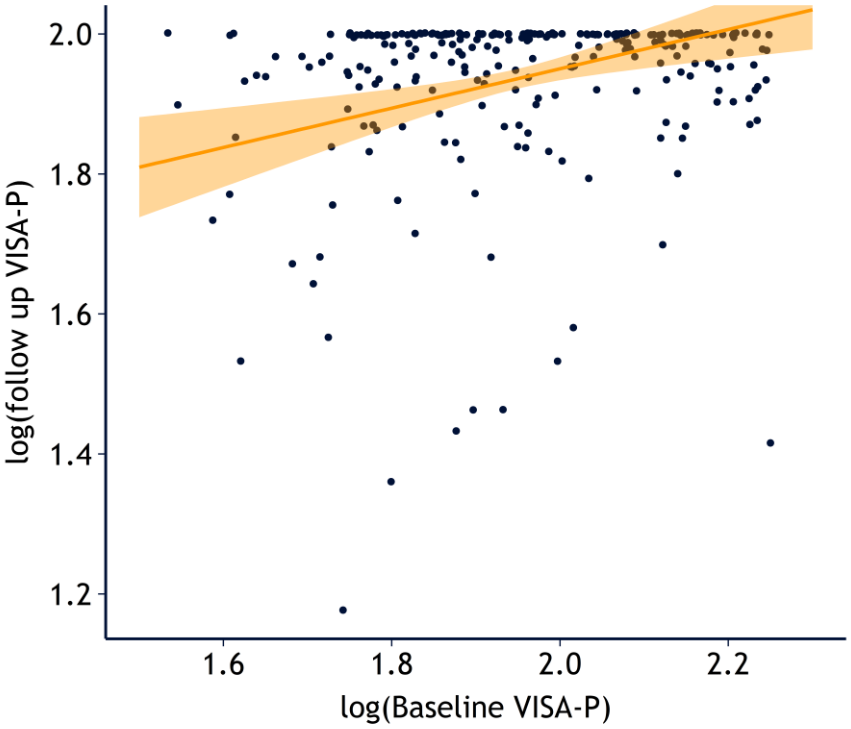

Figure A2. The correlation between VISA-P at baseline and VISA-P at follow up, transformed by the 10th logarithm to handle non-normality. The data points are the true observations. The yellow line is the predicted value from a linear model, with 95% heteroskedasticity-robust confidence intervals. Log-transformed Baseline VISA-P was significantly correlated with follow up VISA-P, but with broad confidence intervals (β=0.28, 95% cluster-robust CI = 0.06–0.50, p = 0.011). Of 276 knees in total, 8 missing baseline VISA-P and 1 missing follow up VISA-P were removed from the analysis (n=267).

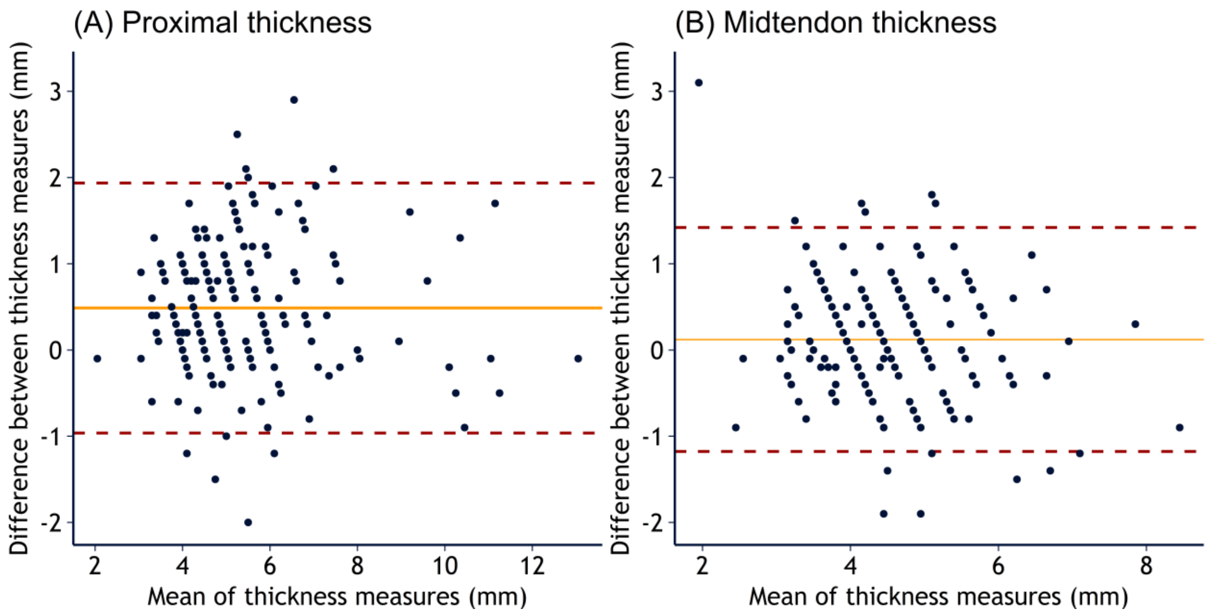

Figure A3. Bland-Altman plot comparing the difference between the two radiologists' thickness measures (y-axis) and the mean thickness between the two radiologists' measures (x-axis). The yellow lines represent the mean difference in millimeters between radiologists at 0.49 (95% CI=-0.96–1.94) proximally and 0.12 (95% CI=-1.18–1.42) in the midtendon, and the red dashed lines represent the confidence intervals. Based on 192 knees with thickness measures.

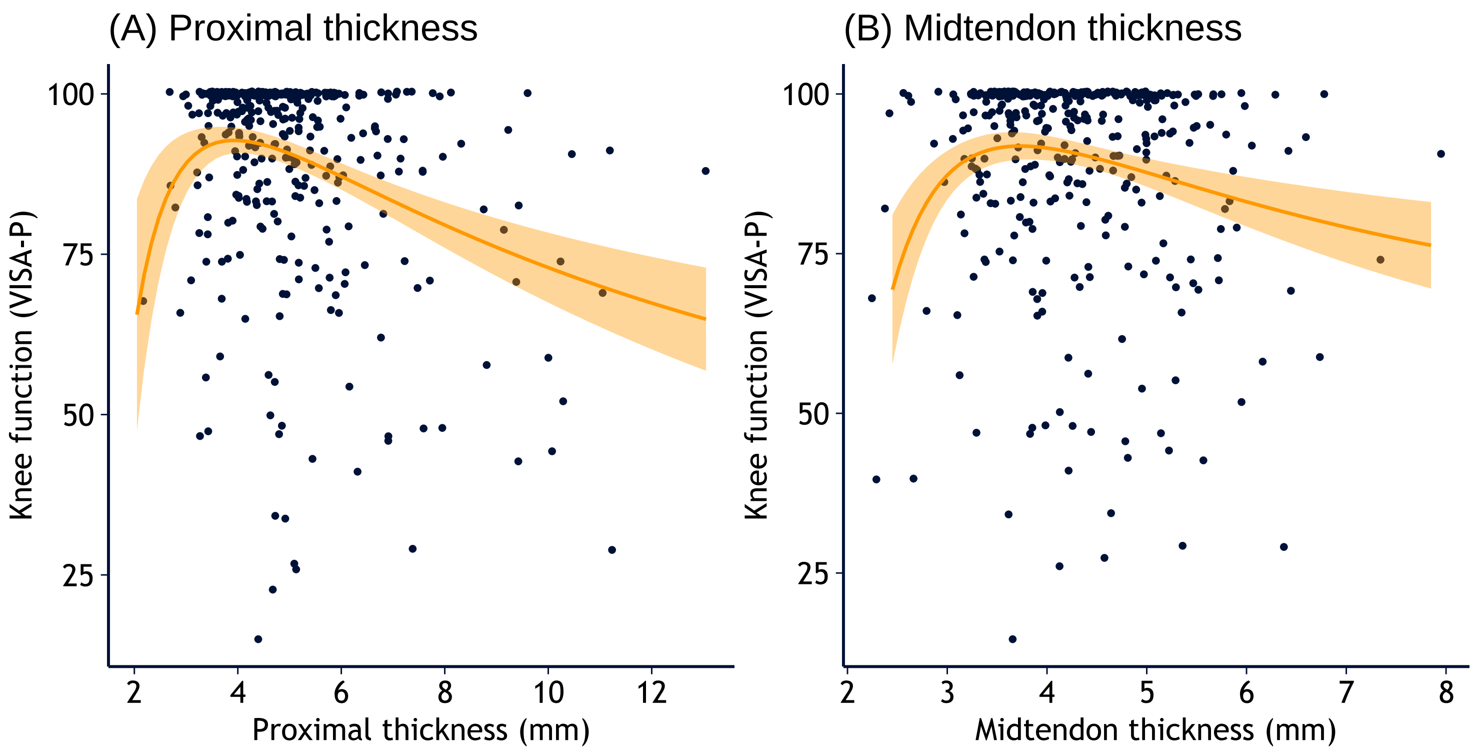

Figure A4. The relationship between tendon thickness and knee function, for (A) proximal thickness ($p < 0.001$), and (B) midtendon thickness ($p < 0.001$). The data points are the true observations. The yellow line is the predicted value from fractional polynomial models, with 95% confidence bands. Adjusted for timepoint (t), which could either be 0 for baseline (ultrasound) or 1 for follow up (MRI), the final models were:

(A) $VISA\text{-}P = -55 + -60x^{-1} + 189x^{-0.5} + -1.7t$ where $x = \frac{proximal\ thickness\ (mm)}{10}$, and

(B) $VISA\text{-}P = 58 + 14x^{-2} + (9.7x^{-2} * log\ [x]) + -1.9t$ where $x = \frac{midtendon\ thickness\ (mm)}{10}$.

The most optimal polynomial fit was chosen based on a form of backwards stepwise deletion estimated by the mfp R package.[1] One outlier had extreme levels of dfBeta and set to a less extreme value to avoid biasing the regression.

Tables

Table A1. Interrater reliability of categorical variables between two radiologists examining MRI scans.

Variable	Cohen's Kappa	CI
Increased interstitial signals	0.46	0.28–0.63
Interstitial ruptures	0.62	0.38–0.85
Partial ruptures of tendon insertion	0.43	0.24–0.63
Focal tendon thickness	0.52	0.33–0.71

Abbreviations: CI = 95% Confidence Interval

Table A2. Association of jumper's knee at baseline with clinical outcomes at 11-year follow up, adjusted for biological sex.

**Outcome: VISA-P score at follow-up (n = 275 knees)[1]**	**β**	**SE**	**95% CI**	**p**
Intercept	90	1.836	86–94	< 0.001
Baseline jumper's knee				
No (reference)	-	-	-	-
Yes	-9.3	3.891	-16.9–-1.7	0.017
Biological sex				
Female	-	-	-	-
Male	0.01	3.044	-5.9–6.0	0.996
**Outcome: IKDC at follow-up (n = 138 participants)**				
Intercept	92	1.085	90–95	< 0.001
Baseline jumper's knee				
No (reference)	-	-	-	-
Yes	-11	2.521	-15.4–-5.5	< 0.001
Biological sex				
Female	-	-	-	-
Male	0.2	1.844	-3.4–3.8	0.906

Abbreviations: CI = 95% cluster-robust Confidence Interval; IKDC = International Knee Documentation Committee Subjective Knee Form; SE = Standard Error; VISA-P = Victorian Institute of Sport Assessment score

[1]One knee had missing data on follow-up VISA-P.

Statistical model specification

Change between baseline and follow-up clinical outcomes

To test whether jumper's knee at baseline was associated with reduced knee function 11 years later, a linear regression model was run. The independent variable was the baseline status of the knee (jumper's knee/no jumper's knee), and the outcome was the VISA-P score at follow up (0–100). A Directed acyclic graph was drawn based on our hypotheses to determine whether any confounder adjustment was necessary.[2] Biological sex was identified as a confounder, and included in the model as an independent variable. Another linear regression model was run with the same setup, where IKDC score was the outcome instead.

Change between baseline and follow-up MRI outcomes

To test whether abnormal tendons at baseline normalized over the follow up period, a one-tailed binomial test was run, with 95% Wilson binomial confidence intervals.[3] In addition, we tested whether tendon thickness changed between baseline and follow up. The difference in proximal and mid portion thickness between baseline and follow up was calculated. A one-sample t-test was conducted to see if the difference (change from baseline) differed from 0, for those with pathological and non-pathological tendons at baseline, respectively.

Follow-up outcomes only

A linear model was run to see if pathological versus non-pathological tendons at follow up differed in VISA-P scores. Knee condition (ACL injury, meniscus injury or cartilage injury) was adjusted for as a confounder.

To determine the association between tendon thickness and knee function, two linear models were run with VISA-P as the outcome, and proximal and mid portion thickness as the respective independent variables. Fractional polynomials were added to explore non-linearity between these continuous variables.[4] Overly influential values were checked with dfBeta, and if they skewed the model to an unreasonable degree, they were *not removed*, but set to a less extreme value in accordance with Harrell Jr [5].

Assumptions

We assumed within-individual correlations between knees in the same individual. In addition, assumptions of normality and homoscedasticity of residuals were violated in models using follow up VISA-P. To account for all these assumptions, we estimated heteroskedasticity and cluster-robust standard errors, confidence intervals and p-values in all tests.[6]

When calculating Intraclass Correlation Coefficient (IRR), the option "single fixed raters" was used in the psych R package,[7] assuming that raters were fixed effects.

References

1. mfp: Multivariable Fractional Polynomials [program]. 1.5.2 version: The Comprehensive R Archive Network, 2015.
2. Stovitz SD, Shrier I. Causal inference for clinicians. *BMJ evidence-based medicine* 2019 doi: 10.1136/bmjebm-2018-111069

3. Brown LD, Cai TT, DasGupta A. Interval Estimation for a Binomial Proportion. *Statistical Science* 2001;16(2):101-33, 33.
4. Bache-Mathiesen LK, Andersen TE, Dalen-Lorentsen T, et al. Not straightforward: modelling non-linearity in training load and injury research. *BMJ Open SEM* 2021;7(3):e001119. doi: 10.1136/bmjsem-2021-001119
5. Harrell Jr FE. Regression modeling strategies. *BIOS* 2017;330:2018.
6. Pustejovsky JE, Tipton E. Small-Sample Methods for Cluster-Robust Variance Estimation and Hypothesis Testing in Fixed Effects Models. *Journal of Business & Economic Statistics* 2018;36(4):672-83. doi: 10.1080/07350015.2016.1247004
7. Revelle W. psych: Procedures for Psychological, Psychometric, and Personality Research [v. 2.3.12]: Northwestern University; 2023 [Available from: https://CRAN.R-project.org/package=psych.
